# Involvement of neutrophil extracellular traps in the pathogenesis of glomerulonephritis in a case of systemic lupus erythematosus and antineutrophil cytoplasmic antibody-associated vasculitis overlap syndrome

**DOI:** 10.1007/s13730-021-00682-y

**Published:** 2022-01-13

**Authors:** Arisa Senda, Ryutaro Sasai, Kurumi Kato, Yuka Nishibata, Sakiko Masuda, Akihiro Ishizu, Noriko Takahara

**Affiliations:** 1 Department of Internal Medicine, Ako Municipal Hospital, 1090 Nakahiro, Ako, Hyogo 678-0232 Japan; 2 Department of Medical Laboratory Science, Faculty of Health Sciences, Hokkaido University, Kita-12, Nishi-5, Kita-ku, Sapporo, Hokkaido 060-0812 Japan

**Keywords:** Systemic lupus erythematosus, Neutrophil extracellular traps, ANCA-associated vasculitis

## Abstract

Systemic lupus erythematosus (SLE) and antineutrophil cytoplasmic antibody-associated vasculitis (AAV) are autoimmune diseases that often cause rapidly progressive glomerulonephritis, with neutrophil extracellular traps (NETs) involved in their pathogenesis. However, the involvement of NETs in the renal damage caused by SLE/AAV overlap syndrome has not been clarified yet. In this study, we detected renal deposition of NETs in a patient with SLE/AAV overlap syndrome. In addition, a significantly increased level of NET-inducing activity was observed in the patient’s serum, which improved with treatment. On the other hand, a markedly lower level of NET degradation was observed in the patient’s serum as compared to healthy subjects’ sera, without any posttreatment changes. These findings suggest that NETs may play a role in the pathogenesis of renal injury associated with SLE/AAV overlap syndrome.

## Introduction

Systemic lupus erythematosus (SLE) and antineutrophil cytoplasmic antibody (ANCA)-associated vasculitis (AAV) are autoimmune diseases that often cause rapidly progressive glomerulonephritis (RPGN). In SLE, excessively produced autoantibodies—especially anti-deoxyribonucleic acid (DNA) antibodies—bind to their respective antigens (e.g., DNA fibers) to form immune complexes, which are deposited in the kidneys and other organs, and subsequently induce inflammation through activation of the complement system [1].

On the other hand, AAV is characterized by inflammation of small blood vessels, mainly affecting the kidneys and lungs [2]. In AAV, myeloperoxidase (MPO) and proteinase 3 (PR3), which are proteins mainly expressed in neutrophil granules, develop antigenicity, leading to ANCA production. Neutrophils activated by ANCA binding can then infiltrate into the local glomerular area, causing necrotizing crescentic glomerulonephritis.

Recently, it has been suggested that neutrophil extracellular traps (NETs) are associated with a variety of autoimmune diseases, such as SLE and AAV. NETs are extracellular DNA structures that are involved in a powerful immune mechanism whereby neutrophils capture and kill microorganisms by releasing bactericidal proteins and DNA fibers into the extracellular space [3]. It has been suggested that dysregulation in NET formation has an important role in the production of anti-DNA antibodies and myeloperoxidase-antineutrophil cytoplasmic antibody (MPO-ANCA) [4, 5]. Furthermore, it has been shown that sera from SLE and AAV patients induce NETs in healthy neutrophils, but the degradation rate of NETs is significantly lower than that of healthy sera [6]. Thus, evaluating serum NET induction and degradation activities is helpful in understanding the NET-mediated pathology underlying SLE and AAV.

Here, we report the case of RPGN in a patient with SLE/AAV overlap syndrome. The involvement of NETs in the renal damage caused by SLE/AAV overlap syndrome has not been reported previously. Further, we investigated (1) NET deposition in the patient’s renal tissue and (2) NET-inducing and NET-degrading activities in the patient’s serum as well as their posttreatment changes.

## Case report

**Fig. 1:**
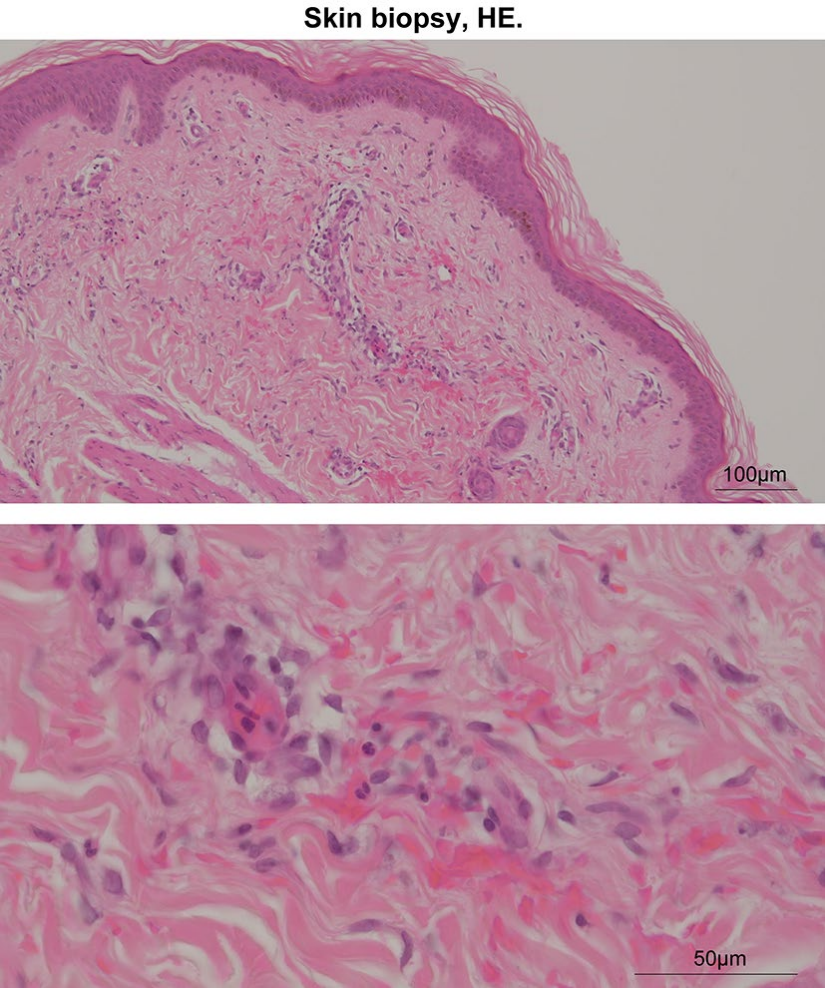
Skin biopsy findings (hematoxylin and eosin). A basket-weave pattern of the epidermis was observed. Infiltration of inflammatory cells, including neutrophils, with nuclear dust around the vessels in the upper dermis and extravasation of red blood cells were observed

A 36-year-old woman presented to our hospital with a fever of 39 °C for a month, dry cough, redness in both eyes, polyarthralgia, purpura, subcutaneous bleeding, edema of the lower legs, nonscarring alopecia, and stomatitis. Laboratory findings were remarkable for proteinuria, hematuria, elevated serum creatinine concentration (estimated glomerular filtration rate of 59.0 mL/min/1.73 m^2^), MPO-ANCA > 300 IU/mL, low complement levels, and positive anti-double-stranded DNA (anti-dsDNA) antibody (Table 1). A biopsy specimen was obtained from the rash on the lower leg. Perivascular infiltration of inflammatory cells, including neutrophils, with nuclear dust around the vessels in the upper dermis, which was consistent with leukocytoclastic vasculitis, was noted (Fig. 1).

**Table 1:** Laboratory findings on admission

Peripheral blood		Blood chemistry		Serology	
RBC	330 × 10^4^/μL	CRP	2.46 mg/dL	ANA	640 times
Hb	10.3 g/dL	TP	7.7 g/dL	Anti-dsDNA antibody	125 IU/mL
Ht	30.3%	CK	74 U/L	RF	7 IU/mL
Plt	36.0 × 10^4^/μL	T-Bil	0.5 mg/dL	aCL-IgG	13 U/mL
WBC	4200/μL	AST	56 U/L	C3	46 mg/dL
Neu	79.5%	ALT	31 U/L	C4	7 mg/dL
Mono	1.1%	LDH	314 U/L	CH50	15.5 U/L
Lympho	15.2%	Uric acid	4.8 mg/dL	MPO-ANCA	> 300.0 IU/mL
Eosino	1.0%	BUN	14 mg/dL	PR3-ANCA	1 IU/mL
Baso	2.0%	Cr	0.88 mg/dL		
		Na	133 mEq/L	Urinalysis	
		K	4.5 mEq/L	RBC	300–599/HPF
		Cl	99 mEq/L	WBC	5–9/HPF
		Ca	8.6 mg/dL	Protein/Cr	1.09 g/g-Cr

*RBC* red blood cell, *Hb* hemoglobin, *Ht* hematocrit, *Plt* platelet, *WBC* white blood cell, *Neu* neutrophil, *Mono* monocyte, *Lympho* lymphocyte, *Eosino* eosinophil, *Baso* basophil, *CRP* C-reactive protein, *TP* total protein, *CK* creatinine kinase, *T-Bil* total bilirubin, *AST* aspartate aminotransferase, *ALT* alanine aminotransferase, *LDH* lactate dehydrogenase, *BUN* blood urea nitrogen, *Cr* creatinine, *Na* sodium, *K* potassium, *Cl* chloride, *Ca* calcium, *ANA* anti-nuclear antibody, *Anti-dsDNA* anti-double stranded deoxyribonucleic acid, *RF* rheumatoid factor, *aCL-IgG* anti-cardiolipin immunoglobulin G, *C3* complement3, *C4* complement4, *CH50* 50% hemolytic complement activity, *MPO-ANCA* myeloperoxidase-anti-neutrophil cytoplasmic antibody, *PR-ANCA* proteinase-3-anti-neutrophil cytoplasmic antibody, *HPF* high power field

**Fig. 2:**
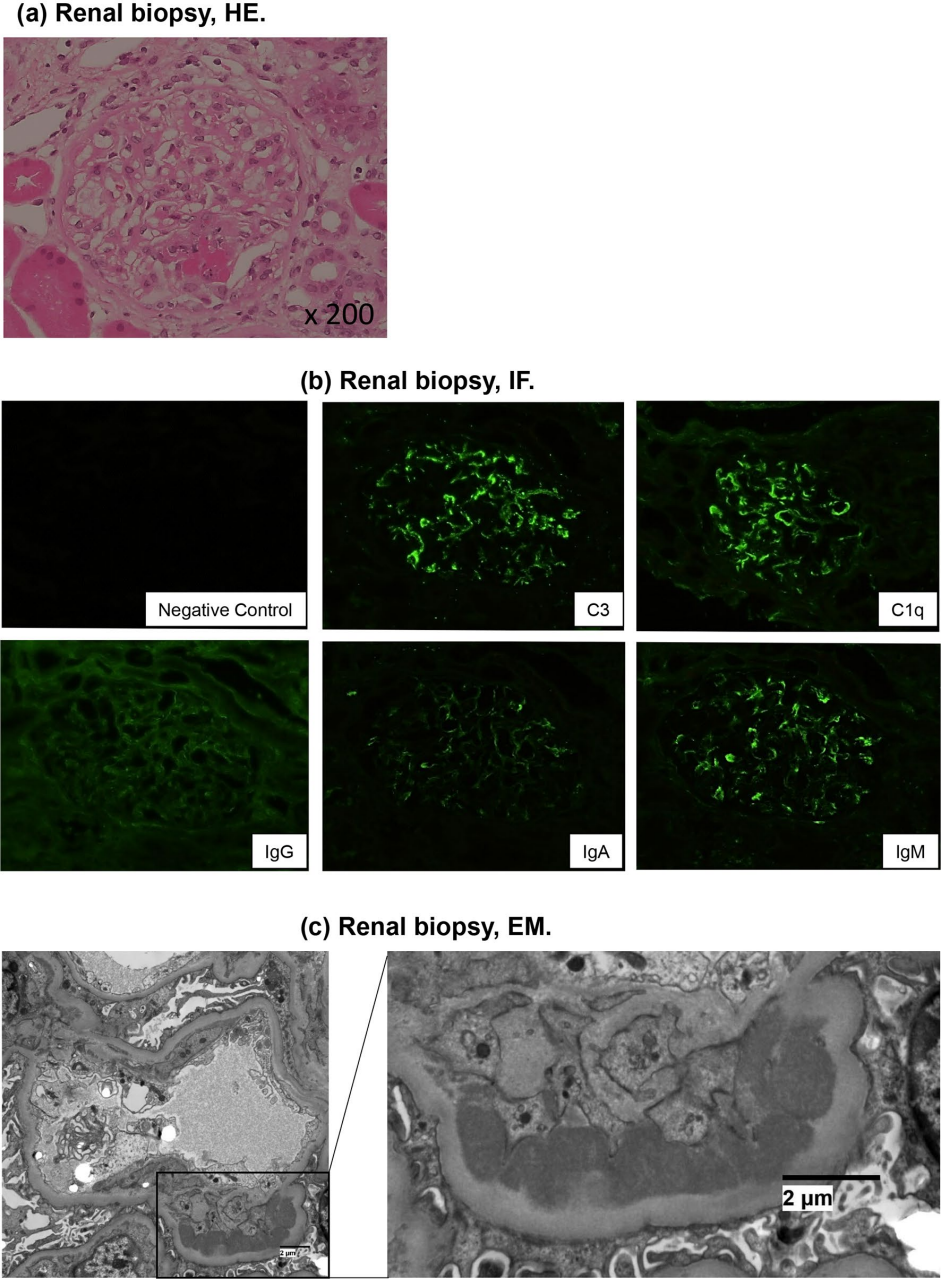
Renal biopsy findings. **a** Fibrinoid necrosis, hematoxylin and eosin staining, **b** immunofluorescence staining, and **c** electron-dense deposits in the subendothelium, electron microscopy

Renal biopsy revealed that a total of 44 glomeruli, including 8 with cellular crescent, 3 with fibrinoid necrosis, and 2 with endocapillary hypercellularity but no global sclerosis, were observed. Focal interstitial inflammation with edema was also observed, although there was no overt peritubular capillaritis (Fig. 2a). Immunofluorescence staining showed granular depositions of IgG(±), IgA(1+), C3(1+), C1q(1+) and IgM(1+) along the capillary wall and partly in the mesangial region (Fig. 2b). Electron microscopy showed dense deposits in the subendothelial and mesangial regions, but no fingerprinting (Fig. 2c). Based on these findings, the patient met the 2019 European League Against Rheumatism (EULAR)/American College of Rheumatology (ACR) classification criteria for SLE diagnosis and was diagnosed as having SLE with type III lupus nephritis (LN). In addition, the patient had a high serum MPO-ANCA level and leukocytoclastic vasculitis in the skin; hence, SLE/AAV overlap syndrome was suspected.

**Fig. 3:**
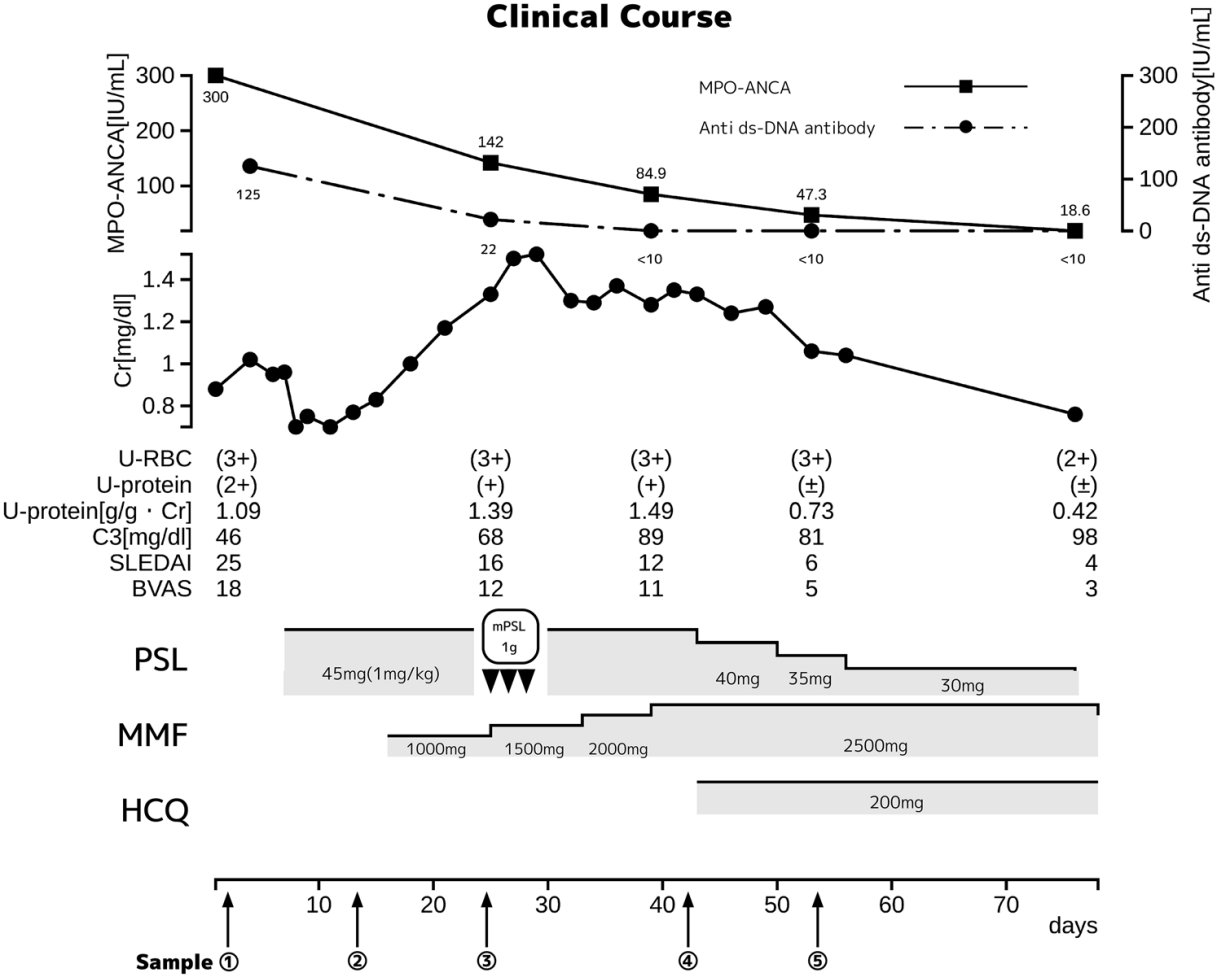
Clinical course. Serum samples were collected at the following time points: ① on admission, ② 8 days after starting treatment with PSL, ③ 10 days after initiating MMF administration, ④ 14 days after starting mPSL administration, ⑤ 10 days after initiating HCQ therapy (*mPSL* methylprednisolone, *PSL* prednisolone, *MMF* mycophenolate mofetil, *HCQ* hydroxychloroquine). *Cr* creatinine, *u-RBC* urinary red blood cells, *u-protein* urinary protein, *C3* complement 3, *SLEDAI* Systemic Lupus Erythematosus Disease Activity Index, *BVAS* Birmingham Vasculitis Activity Score.

After renal biopsy, on post-admission day (PAD) 3, oral administration of prednisolone (PSL) 1 mg/kg/day was started. The patient’s symptoms such as fever and skin rash improved upon treatment with PSL, but her renal function deteriorated further. On PAD 13, 1 g/day of mycophenolate mofetil (MMF) was added to the therapeutic regimen, followed by gradual dose increments. On PAD 25, methylprednisolone was also administered at 1 g/day for 3 days, whereupon her renal function gradually improved. On PAD 41, 200 mg/day of hydroxychloroquine (HCQ) was added. With this series of treatments, clinical scores such as SLE Disease Activity Index (SLEDAI) and Birmingham Vasculitis Activity Score (BVAS) improved and anti-dsDNA antibody and MPO-ANCA levels significantly decreased, but the decrease in MPO-ANCA levels was slower than the decrease in anti-dsDNA antibody levels (Fig. 3). On PAD 40, anti-dsDNA antibody and C3 levels normalized, but MPO-ANCA levels, serum creatinine levels, and proteinuria did not improve sufficiently. MPO-ANCA remained weakly positive at 3.6 IU/mL, even after 6 months. Based on this discrepancy between anti-dsDNA antibodies and MPO-ANCA in response to the therapy, we considered that AAV overlapped with SLE in this patient.

### NET deposition in renal tissue

**Fig. 4:**
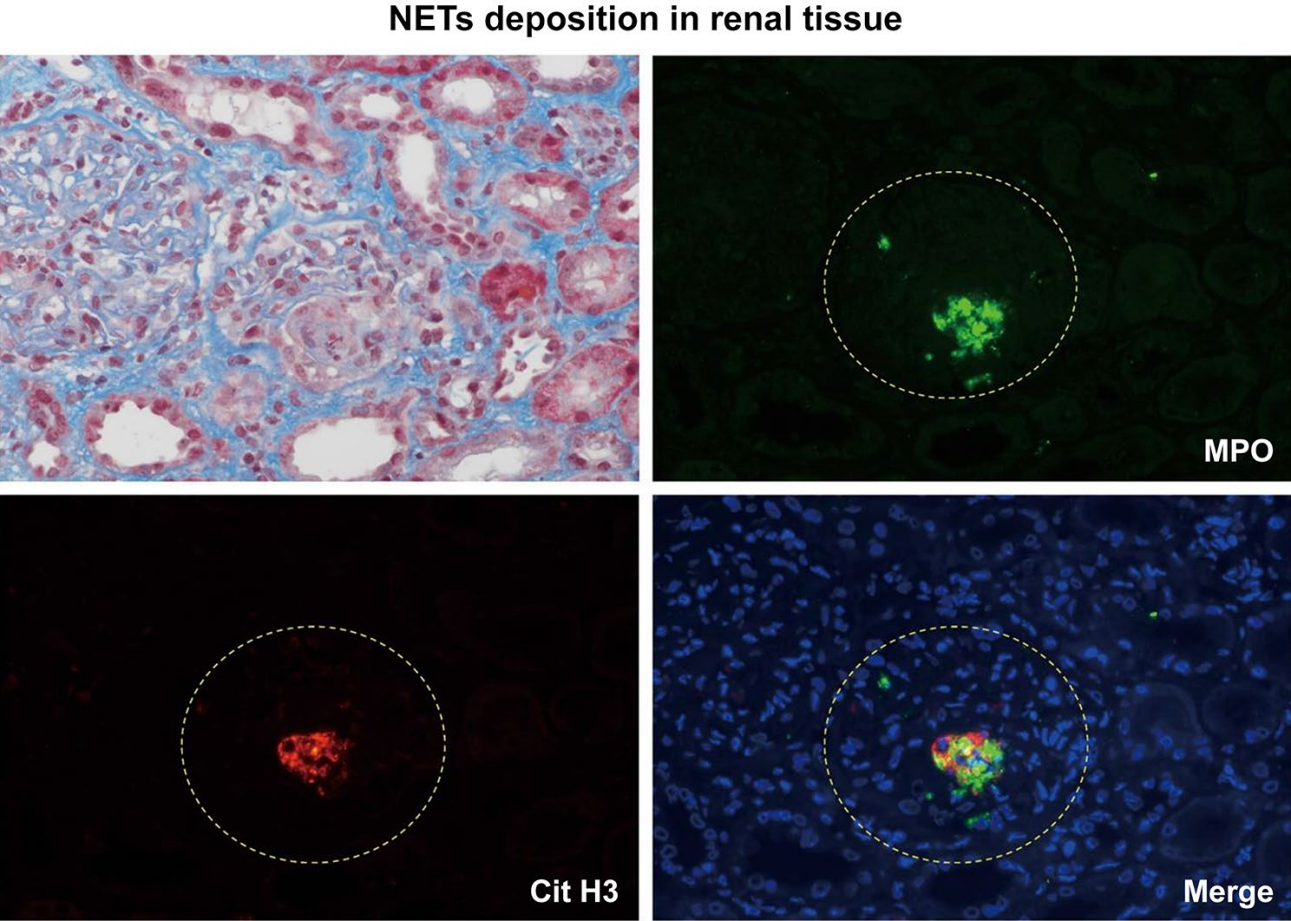
Immunofluorescent findings in the kidney. Immunofluorescent findings showing the colocalization of myeloperoxidase (MPO) and citrullinated histone H3 (Cit H3) within fibrinoid necrosis in the glomeruli, confirming the deposition of neutrophil extracellular traps. Original magnification, × 400

To clarify the involvement of NETs in the pathogenesis of renal damage, we investigated the deposition of NETs in the patient’s renal tissue. Immunofluorescence analyses were performed as previously reported [7]. In brief, citrullinated histone H3 (Cit-H3) was stained using anti-Cit-H3 antibody (Abcam, ab5103); DNA, using 4′6-diamidino-2-phenylindole (DAPI; Sigma-Aldrich, St. Louis, MO); and MPO, using anti-MPO antibody (R&D Systems, AF3776) to visualize NETs. Merged images of the stained, formalin-fixed, paraffin-embedded sections of the patient’s renal tissue showed colocalization of MPO and Cit-H3 within fibrinoid necrosis in the glomeruli, confirming the deposition of NETs (Fig. 4). The deposition of NETs was also observed in the tubular interstitial space focally.

### NET-inducing and NET-degrading activities

Serological analyses were approved by the Ethical Committee of Ako Municipal Hospital (Permission No. 2020-0004). After obtaining written informed consent from the patient, we collected serum samples at the following time points: ① on admission, ② 8 days after starting treatment with PSL 45 mg/day (1 mg/kg/day), ③10 days after initiating MMF administration, ④ 14 days after starting methylprednisolone administration, and ⑤ 10 days after initiating HCQ therapy (Fig. 3).

First, we examined whether the patient’s sera could induce NETs, as described previously [6]. Neutrophils were extracted from the peripheral blood samples of healthy volunteers. The neutrophils were seeded on chamber slides (1 × 10^6^/mL), incubated at 37 °C for 30 min, pretreated with or without 5 ng/mL TNF-α for 15 min, and then incubated with 10% patient’s serum. After incubation at 37 °C for 4 h, the supernatant was removed, and the remaining cells on the slide were fixed with 4% paraformaldehyde and sealed with DAPI-containing mounting agent. Photographs of random fields were obtained using a fluorescence microscope (magnification 200× or 400×), NET formation in each sample was assessed as DAPI-positive extracellular chromatin fibers using ImageJ software (NIH, Bethesda, MD, USA), and NET area/neutrophil count was quantified.

**Fig. 5:**
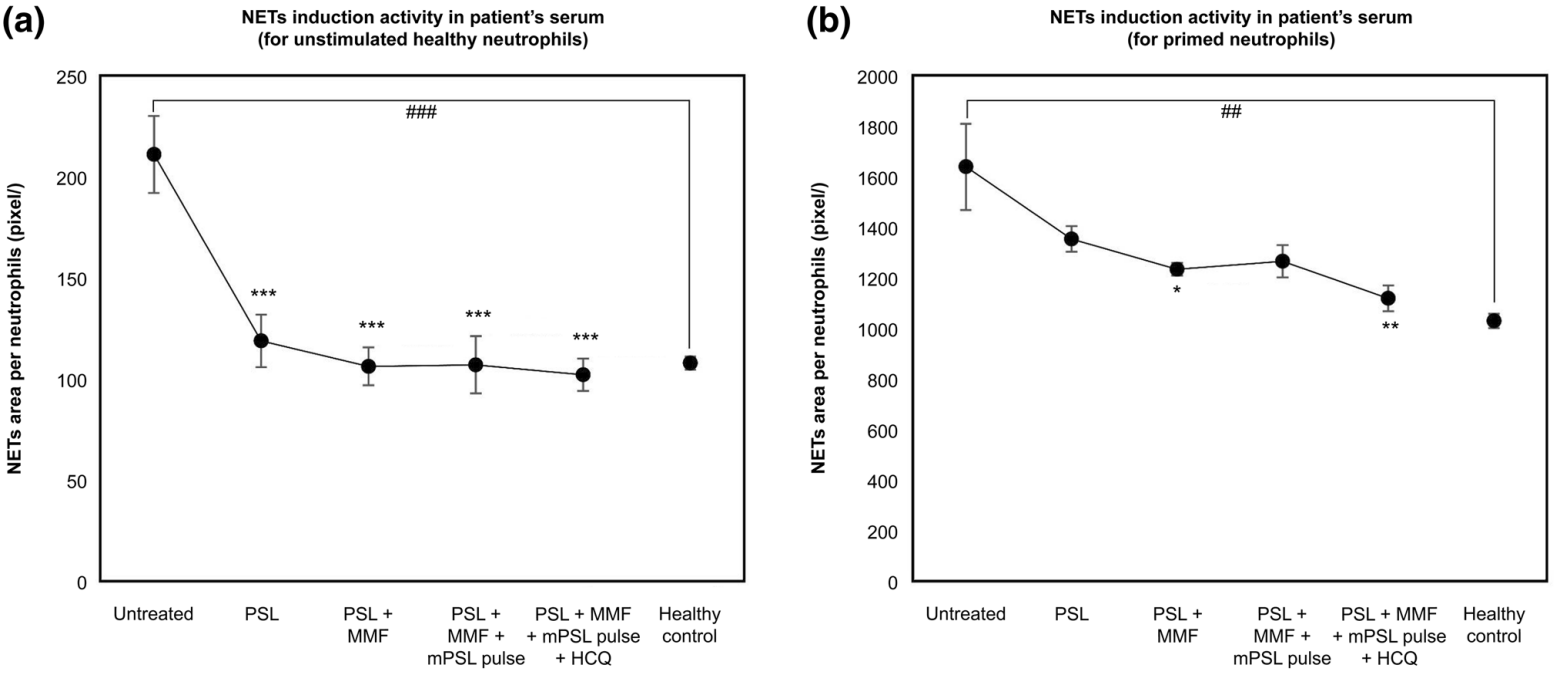
Neutrophil extracellular traps (NETs) induction activity in patient’s serum. The activities were examined against **a** unstimulated and **b** primed neutrophils. Data are shown as the mean ± SD of four to six samples. *p < 0.05, **p < 0.01, ***p < 0.001 vs. untreated, ^##^*p* < 0.01, ^###^*p* < 0.001 in pairwise comparison using *t* tests (Bonferroni correction). *NET* neutrophil extracellular trap, *PSL* prednisolone, *MMF* mycophenolate mofetil, *mPSL* methylprednisolone, *HCQ* hydroxychloroquine

Patient’s sera collected at untreated time points (①) showed high NET-inducing activity against unstimulated neutrophils from healthy subjects (Fig. 5a); this activity was markedly reduced when PSL was administered at 45 mg/day. With further intensification of the treatment (②–⑤), the NET-inducing activity decreased to the same level as in healthy subjects. Moreover, sera from untreated patients also showed significantly higher NET-inducing activity against neutrophils from healthy subjects primed with TNF-α (Fig. 5b). In contrast to the results for unstimulated neutrophils, the treatment-induced improvement in NET-inducing activity for neutrophils primed with TNF-α was less evident.

**Fig. 6:**
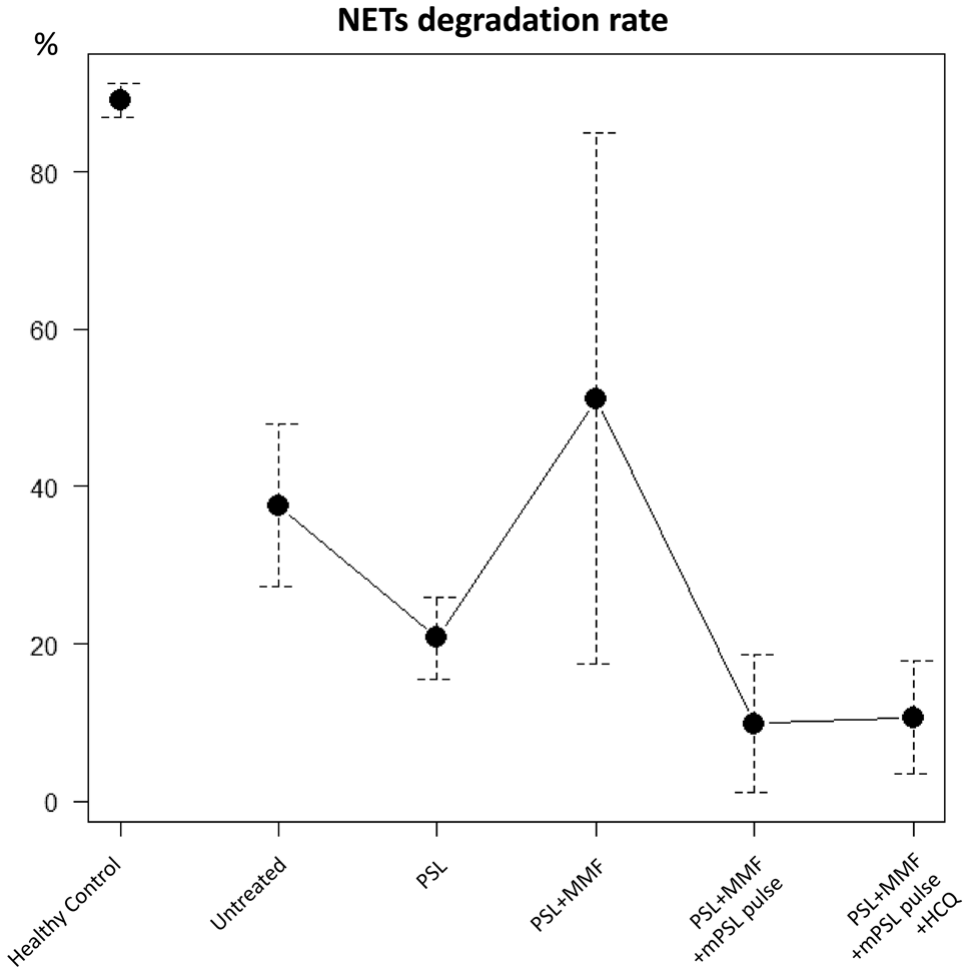
Neutrophil extracellular traps (NETs) degradation rate. Data are shown as the mean ± SD of five to seven samples

As previously reported, we also examined the NET-degrading activity in the serum of this patient [6]. Peripheral blood neutrophils from healthy volunteers were seeded into slide chambers (1 × 10^6^/mL), incubated at 37 °C for 30 min, and then reacted with 100 nM phorbol myristate acetate (Sigma-Aldrich, St. Louis, MO) for 3 h at 37 °C. The cells were washed with phosphate-buffered saline (PBS), following which 10% serum sample (①–⑤) was added and incubated at 37 °C for 6 h. After washing again with PBS, the cells remaining on the slide were fixed with 4% paraformaldehyde and mounted with a DAPI-containing solution. Photographs of random fields were obtained using a fluorescence microscope (magnification 200×), and the residual NETs area was determined using ImageJ software (6 fields/chamber slide). NETs degradation rate (%) was calculated as follows: [(residual NETs incubated with PBS − residual NETs incubated with serum)/residual NETs incubated with PBS] × 100. The NET-degrading activity of the patient’s serum before treatment initiation was found to be lower than that of the healthy controls’ sera (Fig. 6). Furthermore, there was no change in the NET-degrading activity with treatment.

## Discussion

The case presented herein was diagnosed as an SLE/AAV overlap syndrome causing RPGN, with NET deposition observed at the site of fibrinoid necrosis in the glomeruli. Besides, the NET-inducing activity in the patient’s serum, which was significantly high, improved with treatment.

The patient was diagnosed with SLE based on the 2019 EULAR/ACR classification criteria for SLE. A renal biopsy revealed type III LN with multiple immune complex deposits. In addition, the patient had very high serum MPO-ANCA levels (> 300 IU/mL) and leukocytoclastic vasculitis in the skin, leading to the suspicion of an overlap with AAV. The concept of SLE/AAV overlap syndrome is a rare condition that was proposed in 2008 [8]. Although this case met the criteria for SLE, the diagnosis of AAV was based solely on our findings (i.e., elevated MPO-ANCA level, purpura, and subcutaneous bleeding), which did not fully meet the AAV definitions presented by the Japan Research Committee of the Ministry of Health, Labor, and Welfare. However, according to previous reports, patients with SLE/AAV overlap syndrome do not always meet the diagnostic criteria for both, and one or the other feature may be more prominent [9]. In some cases, the symptoms of both diseases may present at different times. On the other hand, it has already been reported that 16–42% of SLE cases and 37–53% of LN cases are ANCA positive [10]. LN patients with ANCA positivity tend to have a higher clinical severity than those without ANCA. These findings suggest that in patients with SLE/AAV overlap syndrome, these two diseases may not only coexist but also interact to exacerbate the condition.

Although the involvement of NETs in the pathogenesis of LN and AAV-related nephritis has been reported recently [11], the significance of NETs in renal damage secondary to SLE/AAV overlap syndrome remains to be elucidated. Scattered images taken after immunofluorescence staining of the patient’s renal biopsy sections demonstrated colocalization of MPO and Cit-H3 within impaired glomeruli, confirming deposition of NETs.

NET formation (NETosis) by neutrophils is a crucial mechanism for innate immunity [3]. Neutrophils attack pathogens either directly or by releasing NETs, which contain nuclear materials such as DNA and MPO, into the extracellular space. Overproduction of NETs and the persistence of undegraded NETs may further promote the production of autoantibodies against dsDNA fibers and MPO, leading to a vicious cycle and a self-perpetuating condition. NETs are not only immunogenic, but also cytotoxic by nature. Murine studies have shown that NET-derived histones can cause crescentic glomerulonephritis [12]. It has been demonstrated that extracellular MPO, a component of NETs, is involved in oxidative stress production in AAV-related nephritis [13]. NETs present in the kidney are known to be associated with the degree of proteinuria and to drive glomerular endothelial-to-mesenchymal transition in patients with SLE [14]. Here, the deposition of NETs coincided with the site of glomerular necrosis or destruction, suggesting that NETs might have been involved in the development of RPGN in our patient.

Furthermore, we demonstrated that the NET-inducing activity in the patient’s serum was high before therapy initiation and later improved with the treatment. It has been reported that NET-inducing activities in serum are increased in both patients with SLE [15] and AAV [16]. Interestingly, however, various characteristic differences in the ex vivo formation of NETs (including morphology, release kinetics, triggers, and pathways) have been shown to exist between SLE and AAV [17]. Therefore, we examined the NET-inducing activity of SLE/AAV overlap syndrome in patient’s serum in two ways, using both unstimulated and TNF-α-stimulated neutrophils from healthy subjects. MPO appears on the cell surface when neutrophils are stimulated with TNF-α, so using TNF-α-stimulated neutrophils may reflect NET-inducing activity through ANCAs [6]. In the case of unstimulated neutrophils, NET induction is thought to have been triggered by immune complexes and damage-associated molecular patterns in the patient’s serum; this activity was rapidly ameliorated by 1 mg/kg/day of PSL. However, the therapeutic effect of PSL alone on the NET-inducing activity of the patient’s serum against neutrophils stimulated with TNF-α turned out to be insufficient, requiring further immunosuppressive agents and higher doses of PSL. In the present case, the decrease in the NET-inducing activity against unstimulated neutrophils was consistent with the improvement in the clinical markers of SLE, such as anti-dsDNA antibody levels and serum complement titers. In contrast, the decrease in the serum NET-inducing activity against TNF-α-stimulated neutrophils was consistent with the changes in MPO-ANCA levels. Furthermore, ANCA with NET-inducing activity was found in the serum even when anti-ds-DNA antibody and serum C3 were negative. These findings suggest that the NET-inducing activity on unstimulated and TNF-α-stimulated neutrophils reflects the disease activity of SLE and AAV, respectively. Furthermore, we considered that the AAV lesions in this patient may have had a slightly lower initial response to this treatment than the coexisting SLE lesion.

Moreover, the NET-degrading activity in the patient’s serum was low and exhibited no response to the treatment. This was consistent with a previous study demonstrating that NET-degrading activity in the sera of patients with SLE and AAV was low regardless of their disease activity, thereby suggesting it to be the cause rather than the result of the diseases [6].

In conclusion, NETs may be involved in the pathogenesis of renal injury in SLE/AAV overlap syndrome. Standard treatment for LN significantly improved various clinical symptoms, ameliorated renal dysfunction, and decreased autoantibody titers and NET induction activity in the serum. The patients’ clinical course suggests that the NET-inducing activity in the patients’ serum was related to two different pathologies—SLE and AAV.
